# Supplementary material for: Feeding intensity of insect herbivores is associated more closely with key metabolite profiles than phylogenetic relatedness of their potential hosts
Source: PeerJ. 2019 Dec 17;7:e8203. doi: 10.7717/peerj.8203 (PMC6924328; doi:10.7717/peerj.8203)
Supplement: Supplemental Information 1 — Mean values of K values for the association of the feeding intensity by C. cardariae and P. xylostella mapped onto phylograms (trees) based on genetic similarity (chloroplast gene ndhF. or glucosinolate profiles (concentration in μmol/g) of the plant species tested in bioassays. A value of K = 0 indicates no association between the phylogram or dendrogram, while higher values indicate an association is indicated. Compounds detected during GS analysis (n = 45). Compounds in bold (n = 29) were selected for further analysis and for use in generating phylograms. Compounds underlined and nearly co-eluting were collapsed for analysis because they could not be differentiated based on spectra, yielding a final list for analysis (n = 26). Compounds after retention time 30 min were not included in analysis because based on their long retention times these may include GS with further substitutions on the glucose moiety – i.e. R group dimers. RT = Retention Time Figure S1. Dendrograms for species tested based on GS profiles from all leaves, young leaves only (collected above the middle node), and old leaves only (collected below the middle node). [file peerj-07-8203-s001.docx]

**SUPPLEMENTARY TABLES**

| **Table S1** Environmental conditions and experimental design: Temperature (Max and Min °C) and weather (Romandie Meteo) in the afternoon and morning (S = sun, R = rain, C = cloudy, SN = snow) on the dates when the different reps were put outside for a period of 6 to 8 days. Also are given the dates on which the plants in each block (A-E) were placed outside to acclimate, dates on which bioassays were initiated for a period of 48h on these plants, and dates on which when the leaf material for the chemical analysis was sampled. | | | | | | |
| --- | --- | --- | --- | --- | --- | --- |
| Date | T°C day (Max/Min) | Morning Weather | Afternoon Weather | Reps put Outside | Bioassays | Chemical Sampling |
| 31.03 | 9 / 4 | R / S | R / S | A |  |  |
| 1.04 | 13 / 3 | C | C / S | A |  |  |
| 2.04 | 10 / 5 | R | C | A |  |  |
| 3.04 | 7 / 3 | C | R | A |  |  |
| 4.04 | 9 / 3 | C / S | C | A / B |  |  |
| 5.04 | 12 / 2 | C / S | R / S | A / B |  |  |
| 6.04 | 6 / 0 | C / S | C | B |  |  |
| 7.04 | 4 / -4 | S / SN | C / S | B | A |  |
| 8.04 | 4 / -3 | C | C | B | A | A |
| 9.04 | 12 / 3 | C / S | R | B | A |  |
| 10.04 | 12 / 7 | R | R / S | B |  | A |
| 11.04 | 10 / 3 | R / S | R | B |  |  |
| 12.04 | 10 / 1 | C / S | S |  | B |  |
| 13.04 | 14 / 3 | C / S | R / S | C | B |  |
| 14.04 | 10 / 2 | R | R | C | B |  |
| 15.04 | N/A | R | N/A | C |  | B |
| 16.04 | 7 / 0 | S / SN / R | S / SN / R | C |  |  |
| 17.04 | 7 / 0 | C | R | C |  |  |
| 18.04 | 14 / 3 | S | S | C |  |  |
| 19.04 | 14 / 6 | C | S | C |  |  |

| **Table S1 Continued** | | | | | | |
| --- | --- | --- | --- | --- | --- | --- |
| Date | T°C day (Max/Min) | Morning Weather | Afternoon Weather | Reps put Outside | Bioassays | Chemical Sampling |
| 20.04 | 14 / 6 | C | S | C |  |  |
| 21.04 | 11 / 7 | R | R | D |  |  |
| 22.04 | 9 / 7 | R | N/A | D | C | C |
| 23.04 | 11 / 9 | R | R | D | C | C |
| 24.04 | 15 / 7 | C | C | D | C | C |
| 25.04 | 14 / 8 | C | R / S | D |  |  |
| 26.04 | 17 / 5 | S | S | D |  |  |
| 27.04 | 20 / 6 | S | S | D |  |  |
| 28.04 | 15 / 8 | S | R / S | E1 | D |  |
| 29.04 | 14 / 6 | C | N/A | E1 | D | D |
| 30.04 | 12 / 6 | R / S | R | E1 | D |  |
| 1.05 | 14 / 6 | R / S | R / S | E1 |  | D |
| 2.05 | 18 / 4 | N/A | N/A | E1 |  |  |
| 3.05 | 19 / 7 | S | S | E1 |  |  |
| 4.05 | 20 / 8 | S | S | E1 |  |  |
| 5.05 | 18 / 9 | S | S |  | E1 |  |
| 6.05 | 20 / 8 | S | S |  | E1 | E |
| 7.05 | 21 / 9 | S | S | E2 | E1 |  |
| 8.05 | 18 / 9 | S | S | E2 |  |  |
| 9.05 | 21 / 7 | N/A | N/A | E2 |  |  |
| 10.05 | 23 / 12 | N/A | N/A | E2 |  |  |
| 11.05 | 21 / 9 | N/A | N/A | E2 |  |  |
| 12.05 | 20 / 8 | N/A | N/A | E2 |  |  |
| 13.05 | N/A | S | S |  | E2 |  |
| 14.05 | N/A | N/A | N/A |  | E2 |  |
| 15.05 | N/A | N/A | N/A |  | E2 |  |

| **Table S2.** Compounds detected during GS analysis. Of these, 45 are glucosinolates based on fragmentation, and 29 (**in bold**) were selected as a basis for analysis to generate phylograms. Compounds underlined and nearly co-eluting were collapsed for analysis because they could not be differentiated based on spectra, yielding a final list for analysis (*n=26; included in Fig. 1*). Compounds after retention time 30min were not included in analysis because based on their long retention times these may include GS with further substitutions on the glucose moiety – i.e. R group dimers. RT = Retention Time | | | |  |  |
| --- | --- | --- | --- | --- | --- |
| RT (min) | Identification prefix; all are GS unless otherwise noted | | | | |
| 4.6 | | include with elution time 7.8 |  |  |  |
| **7.6** | | **3-(methylsulfinyl) propyl** |  |  |  |
| **7.8** | | **2-hydroxypropyl** |  |  |  |
| **8.0** | | **S-2-hydroxy-3-butenyl or R-2-hydroxy-3-butenyl** |  |  |  |
| **9.6** | | **S-2-hydroxy-3-butenyl or R-2-hydroxy-3-butenyl** |  |  |  |
| 9.7 | | unclear identity as GS |  |  |  |
| **10.2** | | **2-propenyl** |  |  |  |
| **10.5** | | **4-(methylsulfonyl)butyl** |  |  |  |
| **11.4** | | **4-(methylsulfinyl)butyl** |  |  |  |
| **11.4** | | **2-hydroxy-4-pentenyl** |  |  |  |
| 12.5 | | unclear identity as GS |  |  |  |
| **13.6** | | **propyl** |  |  |  |
| **14.0** | | **4-hydroxybenzyl** |  |  |  |
| **14.4** | | **5-(methylsulfinyl) pentyl** |  |  |  |
| **15.2** | | **3-butenyl** |  |  |  |
| **15.6** | | **2-hydroxybenzyl** |  |  |  |
| **16.2** | | **4-hydroxy-3-indolymethyl** |  |  |  |
| **16.2** | | **unclear identity but GS** |  |  |  |
| 17.0 | | unclear identity as GS |  |  |  |
| **17.6** | | **3,4-dihydroxybenzyl** |  |  |  |
| **17.6** | **6-(methylsulfinyl)hexyl** | | | |  |
| **17.6** | **3-methoxybenzyl or 2-methoxybenzyl (isomers)** | | | |  |
| **18.1** | **3-methoxybenzyl or 2-methoxybenzyl (isomers)** | | | |  |
| **18.1** | **butenyl** | | | |  |
| 18.4 | Collapse with elution time 18.1 (3-methoxybenzyl) | | | |  |
| 19.2 | **unknown but clear identification as GS** | | | |  |
| **20.1** | **benzyl** | | | |  |
| **20.7** | **4-methylthiobutyl** | | | |  |
| **21.3** | **3,4-dimethoxybenzyl** | | | |  |
| **21.6** | **indolyl-methyl** | | | |  |
| *22.5* | *4-methoxybenzyl (Internal Standard)* | | | |  |

| **Table S2. Continued** | | |
| --- | --- | --- |
| RT (min) | | Identification prefix – all are GS unless otherwise noted |
| **22.8** | **unknown but clear identification as GS** |  |
| 23.7 | | unclear identification as GS |
| **24.8** | | **2-phenylethyl** |
| **25.0** | | **4-methoxy-3-indolylmethyl** |
| 26.7 | | unclear identity as GS |
| **28.5** | | **9-(methylsulfonyl)nonyl (daughter or parent compound?)** |
| **29.5** | | **9-(methylsulfinyl)nonyl (daughter or parent compound?)** |
| 30.2 | | 10-(methylsulfonyl)decyl |
| 32.3 | | unclear identification as GS |
| 32.9 | | unclear identification as GS |
| 33.1 | | 10-(methylsulfinyl)decyl |
| 34.0 | | unclear identification as GS |
| 35.5 | | unclear identification as GS |
| 36.5 | | unknown but clear identity as GS |
| 37.6 | | MeS-octyl |
| 38.5 | | unknown but clear identification as GS |
| 39.2 | | unclear identification as GS |
| 40.1 | | unknown but clear identification as GS |
| 40.2 | | unclear identification as GS |
| 40.8 | | unclear identification as GS |
| 43 | | unclear identification as GS |
| 44 | | unclear identification as GS |

| **Table S3.** concentration (μmol/g) of GS of the old leaves only of the plant species and populations included in this study. | | | | | |
| --- | --- | --- | --- | --- | --- |
|  | 3-(methylsulfinyl)propyl | 2-hydroxypropyl | 2-(R)-hydroxy-3-butenyl or 2-(S)-hydroxy-3-butenyl | 2-(R)-hydroxy-3-butenyl or 2-(S)-hydroxy-3-butenyl | 2-propenyl |
| *Barbarea orthoceras* | 0.00 | 0.00 | 0.00 | 0.00 | 0.00 |
| *Brassica nigra* | 0.00 | 0.00 | 0.00 | 0.00 | 13.70 |
| *Brassica nigra* (2^nd^ pop) | 0.00 | 0.00 | 0.00 | 0.00 | 21.45 |
| *Camelina microcarpa* | 0.00 | 0.00 | 0.00 | 0.00 | 0.00 |
| *Camelina microcarpa* (2^nd^ pop) | 0.00 | 0.00 | 0.00 | 0.00 | 0.00 |
| *Caulanthus inflatus* | 1.84 | 0.00 | 0.00 | 0.00 | 0.00 |
| *Draba nemorosa* | 0.00 | 0.00 | 0.00 | 0.00 | 0.33 |
| *Hesperis matronalis* | 0.00 | 0.00 | 0.00 | 0.00 | 0.00 |
| *Lepidium campestre* | 0.00 | 0.00 | 0.00 | 0.00 | 0.00 |
| *Lepidium campestre* (2^nd^ pop) | 0.00 | 0.00 | 0.00 | 0.00 | 0.00 |
| *Lepidium crenatum* | 0.00 | 0.00 | 0.00 | 0.00 | 0.00 |
| *Lepidium draba* | 0.00 | 0.00 | 0.00 | 0.00 | 0.00 |
| *Lepidium draba* (2^nd^ pop) | 0.00 | 0.00 | 0.00 | 0.00 | 0.00 |
| *Lepidium latifolium* | 0.00 | 0.00 | 0.00 | 0.00 | 18.10 |
| *Lepidium latifolium* (2^nd^ pop) | 0.00 | 0.00 | 0.00 | 0.00 | 2.04 |
| *Lepidium squamatum* | 0.00 | 0.00 | 0.00 | 0.00 | 0.00 |
| *Lepidium squamatum* (2^nd^ pop) | 0.00 | 0.00 | 0.00 | 0.00 | 0.00 |
| *Stanleya pinnata* | 0.00 | 2.64 | 0.00 | 0.00 | 0.00 |
| *Stanleya viridiflora* | 0.00 | 0.00 | 0.00 | 0.00 | 1.60 |

| **Table S3 Continued** | | | | | |
| --- | --- | --- | --- | --- | --- |
|  | 4-(methylsulfonyl)butyl and 4-(methylsulfinyl)butyl | 2-hydroxy-4-pentenyl | propyl | 4-hydroxybenzyl | 5-(methylsulfinyl)pentyl |
| *Barbarea orthoceras* | 0.00 | 0.00 | 0.00 | 0.59 | 0.00 |
| *Brassica nigra* | 0.00 | 0.00 | 0.00 | 0.81 | 0.00 |
| *Brassica nigra* (2^nd^ pop) | 0.00 | 0.00 | 0.00 | 1.03 | 0.00 |
| *Camelina microcarpa* | 0.00 | 0.00 | 0.00 | 0.65 | 0.00 |
| *Camelina microcarpa* (2^nd^ pop) | 0.00 | 0.00 | 0.00 | 0.77 | 0.00 |
| *Caulanthus inflatus* | 0.00 | 0.00 | 0.00 | 0.94 | 0.00 |
| *Draba nemorosa* | 0.00 | 0.00 | 0.00 | 1.25 | 0.00 |
| *Hesperis matronalis* | 0.67 | 0.00 | 0.00 | 2.46 | 0.00 |
| *Lepidium campestre* | 0.00 | 0.00 | 0.00 | 18.47 | 3.73 |
| *Lepidium campestre* (2^nd^ pop) | 0.43 | 0.00 | 0.00 | 23.32 | 5.06 |
| *Lepidium crenatum* | 0.00 | 0.00 | 5.20 | 1.03 | 0.00 |
| *Lepidium draba* | 9.70 | 0.00 | 0.00 | 16.38 | 0.00 |
| *Lepidium draba* (2^nd^ pop) | 9.45 | 0.00 | 0.00 | 7.80 | 0.00 |
| *Lepidium latifolium* | 0.00 | 0.00 | 0.00 | 0.79 | 0.00 |
| *Lepidium latifolium* (2^nd^ pop) | 0.00 | 0.15 | 0.00 | 0.74 | 0.00 |
| *Lepidium squamatum* | 0.00 | 0.00 | 0.00 | 1.25 | 0.00 |
| *Lepidium squamatum* (2^nd^ pop) | 0.00 | 0.00 | 0.00 | 1.01 | 0.00 |
| *Stanleya pinnata* | 0.00 | 0.00 | 7.49 | 0.91 | 0.00 |
| *Stanleya viridiflora* | 0.24 | 0.00 | 2.04 | 1.99 | 0.00 |

| **Table S3 Continued** | | | | | |
| --- | --- | --- | --- | --- | --- |
|  | 3-butenyl | 2-hydroxybenzyl | 4-hydroxyindol-3-ylmethyl | unknown | 3,4-dihydroxybenzyl |
| *Barbarea orthoceras* | 0.00 | 0.00 | 0.00 | 0.00 | 0.00 |
| *Brassica nigra* | 0.00 | 0.00 | 1.01 | 0.00 | 0.00 |
| *Brassica nigra* (2^nd^ pop) | 0.00 | 0.00 | 1.08 | 0.00 | 0.00 |
| *Camelina microcarpa* | 0.00 | 0.00 | 0.00 | 0.00 | 0.00 |
| *Camelina microcarpa* (2^nd^ pop) | 0.00 | 0.00 | 0.00 | 0.00 | 0.00 |
| *Caulanthus inflatus* | 0.00 | 0.00 | 0.56 | 0.00 | 0.00 |
| *Draba nemorosa* | 0.00 | 0.00 | 1.68 | 0.00 | 0.00 |
| *Hesperis matronalis* | 0.00 | 0.00 | 0.86 | 0.00 | 0.00 |
| *Lepidium campestre* | 0.00 | 0.00 | 0.00 | 0.00 | 0.00 |
| *Lepidium campestre* (2^nd^ pop) | 0.00 | 0.00 | 0.00 | 0.00 | 0.00 |
| *Lepidium crenatum* | 0.00 | 0.05 | 0.00 | 0.00 | 0.00 |
| *Lepidium draba* | 0.00 | 0.00 | 0.06 | 0.00 | 0.00 |
| *Lepidium draba* (2^nd^ pop) | 0.00 | 0.00 | 0.00 | 0.00 | 0.00 |
| *Lepidium latifolium* | 0.00 | 0.00 | 0.00 | 0.00 | 0.00 |
| *Lepidium latifolium* (2^nd^ pop) | 4.80 | 0.00 | 0.00 | 0.00 | 0.00 |
| *Lepidium squamatum* | 0.00 | 0.07 | 0.00 | 1.13 | 0.86 |
| *Lepidium squamatum* (2^nd^ pop) | 0.00 | 0.00 | 0.00 | 0.60 | 0.33 |
| *Stanleya pinnata* | 0.00 | 0.00 | 0.08 | 0.00 | 0.00 |
| *Stanleya viridiflora* | 3.46 | 0.00 | 0.00 | 0.00 | 0.00 |

| **Table S3 Continued** | | | | | |
| --- | --- | --- | --- | --- | --- |
|  | 6-(methylsulfinyl)hexyl | 2-methoxybenzyl and 3-methoxybenzyl | butenyl | benzyl | 4-methylthiobutyl |
| *Barbarea orthoceras* | 0.00 | 45.71 | 0.00 | 0.00 | 0.00 |
| *Brassica nigra* | 0.00 | 0.00 | 0.00 | 0.00 | 0.00 |
| *Brassica nigra* (2^nd^ pop) | 0.00 | 0.00 | 0.00 | 0.00 | 0.00 |
| *Camelina microcarpa* | 0.00 | 0.00 | 0.00 | 0.00 | 0.00 |
| *Camelina microcarpa* (2^nd^ pop) | 0.00 | 0.00 | 0.00 | 0.00 | 0.00 |
| *Caulanthus inflatus* | 0.00 | 0.00 | 0.00 | 0.08 | 0.00 |
| *Draba nemorosa* | 0.00 | 0.00 | 0.00 | 0.00 | 0.00 |
| *Hesperis matronalis* | 0.00 | 0.00 | 0.00 | 0.00 | 0.00 |
| *Lepidium campestre* | 0.19 | 0.00 | 0.00 | 0.00 | 0.00 |
| *Lepidium campestre* (2^nd^ pop) | 0.24 | 0.00 | 0.00 | 0.00 | 0.00 |
| *Lepidium crenatum* | 0.00 | 0.00 | 24.34 | 0.08 | 0.00 |
| *Lepidium draba* | 0.00 | 0.00 | 0.00 | 0.00 | 0.91 |
| *Lepidium draba* (2^nd^ pop) | 0.00 | 0.00 | 0.00 | 0.00 | 0.00 |
| *Lepidium latifolium* | 0.00 | 0.00 | 0.00 | 4.59 | 0.00 |
| *Lepidium latifolium* (2^nd^ pop) | 0.00 | 0.00 | 0.00 | 13.72 | 0.00 |
| *Lepidium squamatum* | 0.00 | 0.00 | 0.00 | 0.07 | 0.00 |
| *Lepidium squamatum* (2^nd^ pop) | 0.00 | 0.00 | 0.00 | 0.00 | 0.00 |
| *Stanleya pinnata* | 0.00 | 0.00 | 0.19 | 0.00 | 0.00 |
| *Stanleya viridiflora* | 0.00 | 0.00 | 1.11 | 0.28 | 0.00 |

| **Table S3 Continued** | | | | | | |
| --- | --- | --- | --- | --- | --- | --- |
|  | 3,4-dimethoxybenzyl | indolyl-methyl | unknown | 2-phenylethyl | 4-methoxyindol-3-ylmethyl | 9-(methylsulfonyl)nonyl and 9-(methylsulfinyl)nonyl |
| *Barbarea orthoceras* | 0.00 | 5.84 | 0.00 | 3.52 | 0.00 | 0.00 |
| *Brassica nigra* | 0.00 | 0.66 | 0.00 | 0.36 | 0.00 | 0.00 |
| *Brassica nigra* (2^nd^ pop) | 0.00 | 1.03 | 0.00 | 2.30 | 0.00 | 0.00 |
| *Camelina microcarpa* | 0.00 | 0.00 | 0.00 | 0.00 | 0.00 | 0.00 |
| *Camelina microcarpa* (2^nd^ pop) | 0.00 | 0.00 | 0.00 | 0.00 | 0.00 | 0.00 |
| *Caulanthus inflatus* | 0.00 | 0.23 | 0.00 | 0.00 | 0.08 | 0.00 |
| *Draba nemorosa* | 0.00 | 0.00 | 0.00 | 0.00 | 0.92 | 6.57 |
| *Hesperis matronalis* | 0.00 | 0.00 | 0.00 | 0.00 | 0.35 | 0.00 |
| *Lepidium campestre* | 0.00 | 0.00 | 0.00 | 0.00 | 0.76 | 0.00 |
| *Lepidium campestre* (2^nd^ pop) | 0.00 | 0.00 | 0.00 | 0.00 | 0.86 | 0.00 |
| *Lepidium crenatum* | 0.00 | 0.00 | 0.00 | 0.00 | 0.00 | 0.00 |
| *Lepidium draba* | 0.00 | 0.00 | 0.00 | 0.00 | 0.77 | 0.00 |
| *Lepidium draba* (2^nd^ pop) | 0.00 | 0.00 | 0.00 | 0.00 | 0.19 | 0.00 |
| *Lepidium latifolium* | 0.00 | 0.00 | 0.00 | 0.00 | 0.26 | 0.00 |
| *Lepidium latifolium* (2^nd^ pop) | 0.00 | 0.00 | 0.00 | 0.00 | 0.55 | 0.00 |
| *Lepidium squamatum* | 0.14 | 0.00 | 8.25 | 0.00 | 0.00 | 0.00 |
| *Lepidium squamatum* (2^nd^ pop) | 0.13 | 0.00 | 7.85 | 0.00 | 0.00 | 0.00 |
| *Stanleya pinnata* | 0.00 | 0.00 | 0.00 | 0.00 | 0.00 | 0.00 |
| *Stanleya viridiflora* | 0.00 | 0.24 | 0.00 | 0.00 | 0.63 | 0.00 |

| **Table S4** Concentration (μmol/g) GS profiles of the young leaves only of the plant species and populations included in this study. | | | | | |
| --- | --- | --- | --- | --- | --- |
|  | 3-(methylsulfinyl)propyl | 2-hydroxypropyl | 2-(R)-hydroxy-3-butenyl or 2-(S)-hydroxy-3-butenyl | 2-(R)-hydroxy-3-butenyl or 2-(S)-hydroxy-3-butenyl | 2-propenyl |
| *Barbarea orthoceras* | 0.00 | 0.00 | 0.00 | 0.00 | 0.00 |
| *Brassica nigra* | 0.00 | 0.00 | 0.00 | 0.00 | 23.03 |
| *Brassica nigra* (2^nd^ pop) | 0.00 | 0.00 | 0.00 | 0.00 | 28.68 |
| *Camelina microcarpa* | 0.00 | 0.00 | 0.00 | 0.00 | 0.00 |
| *Camelina microcarpa* (2^nd^ pop) | 0.00 | 0.00 | 0.00 | 0.00 | 0.00 |
| *Caulanthus inflatus* | 11.31 | 0.00 | 0.00 | 0.00 | 0.83 |
| *Draba nemorosa* | 0.00 | 0.00 | 0.00 | 0.00 | 0.19 |
| *Hesperis matronalis* | 0.00 | 0.00 | 0.00 | 0.00 | 0.00 |
| *Lepidium campestre* | 0.00 | 0.00 | 0.00 | 0.00 | 0.00 |
| *Lepidium campestre* (2^nd^ pop) | 0.00 | 0.00 | 0.00 | 0.00 | 0.00 |
| *Lepidium crenatum* | 0.00 | 0.00 | 0.00 | 0.00 | 0.00 |
| *Lepidium draba* | 0.00 | 0.00 | 0.00 | 0.00 | 0.00 |
| *Lepidium draba* (2^nd^ pop) | 0.00 | 0.00 | 0.00 | 0.00 | 0.00 |
| *Lepidium latifolium* | 0.00 | 0.00 | 0.00 | 0.00 | 24.09 |
| *Lepidium latifolium* (2^nd^ pop) | 0.00 | 0.00 | 0.00 | 0.00 | 6.47 |
| *Lepidium squamatum* | 0.00 | 0.00 | 0.00 | 0.00 | 0.00 |
| *Lepidium squamatum* (2^nd^ pop) | 0.00 | 0.00 | 0.00 | 0.00 | 0.00 |
| *Stanleya pinnata* | 0.00 | 10.87 | 0.00 | 0.00 | 0.00 |
| *Stanleya viridiflora* | 0.00 | 0.00 | 0.94 | 1.09 | 0.63 |

| **Table S4 Continued** | | | | | |
| --- | --- | --- | --- | --- | --- |
|  | 4-(methylsulfonyl)butyl and 4-(methylsulfinyl)butyl | 2-hydroxy-4-pentenyl | propyl | 4-hydroxybenzyl | 5-(methylsulfinyl)pentyl |
| *Barbarea orthoceras* | 0.00 | 0.00 | 0.00 | 0.74 | 0.00 |
| *Brassica nigra* | 0.00 | 0.00 | 0.00 | 1.20 | 0.00 |
| *Brassica nigra* (2^nd^ pop) | 0.00 | 0.00 | 0.00 | 0.69 | 0.00 |
| *Camelina microcarpa* | 0.00 | 0.00 | 0.00 | 0.91 | 0.00 |
| *Camelina microcarpa* (2^nd^ pop) | 0.00 | 0.00 | 0.00 | 0.77 | 0.00 |
| *Caulanthus inflatus* | 0.00 | 0.00 | 0.00 | 0.81 | 0.00 |
| *Draba nemorosa* | 0.00 | 0.00 | 0.00 | 0.91 | 0.00 |
| *Hesperis matronalis* | 0.77 | 0.00 | 0.00 | 2.65 | 0.00 |
| *Lepidium campestre* | 0.10 | 0.00 | 0.00 | 24.12 | 6.46 |
| *Lepidium campestre* (2^nd^ pop) | 1.46 | 0.00 | 0.00 | 18.49 | 6.85 |
| *Lepidium crenatum* | 0.00 | 0.00 | 14.73 | 1.13 | 0.00 |
| *Lepidium draba* | 31.03 | 0.00 | 0.00 | 22.47 | 0.00 |
| *Lepidium draba* (2^nd^ pop) | 26.01 | 0.00 | 0.00 | 17.02 | 0.00 |
| *Lepidium latifolium* | 0.00 | 0.00 | 0.00 | 0.97 | 0.00 |
| *Lepidium latifolium* (2^nd^ pop) | 0.00 | 0.62 | 0.00 | 1.31 | 0.54 |
| *Lepidium squamatum* | 0.00 | 0.00 | 0.00 | 1.51 | 0.00 |
| *Lepidium squamatum* (2^nd^ pop) | 0.00 | 0.00 | 0.00 | 1.05 | 0.00 |
| *Stanleya pinnata* | 0.00 | 0.00 | 30.19 | 1.35 | 0.00 |
| *Stanleya viridiflora* | 0.00 | 0.00 | 7.73 | 1.07 | 0.00 |

| **Table S4 Continued** | | | | | |
| --- | --- | --- | --- | --- | --- |
|  | 3-butenyl | 2-hydroxybenzyl | 4-hydroxyindol-3-ylmethyl | unknown | 3,4-dihydroxybenzyl |
| *Barbarea orthoceras* | 0.00 | 0.00 | 0.00 | 0.00 | 0.00 |
| *Brassica nigra* | 0.00 | 0.00 | 1.22 | 0.00 | 0.00 |
| *Brassica nigra* (2^nd^ pop) | 0.07 | 0.00 | 1.84 | 0.00 | 0.00 |
| *Camelina microcarpa* | 0.00 | 0.00 | 0.00 | 0.00 | 0.00 |
| *Camelina microcarpa* (2^nd^ pop) | 0.00 | 0.00 | 0.00 | 0.00 | 0.00 |
| *Caulanthus inflatus* | 0.00 | 0.00 | 0.68 | 0.00 | 0.00 |
| *Draba nemorosa* | 0.00 | 0.00 | 2.09 | 0.00 | 0.00 |
| *Hesperis matronalis* | 0.00 | 0.00 | 1.64 | 0.00 | 0.00 |
| *Lepidium campestre* | 0.00 | 0.00 | 0.00 | 0.00 | 0.00 |
| *Lepidium campestre* (2^nd^ pop) | 0.00 | 0.00 | 0.05 | 0.00 | 0.00 |
| *Lepidium crenatum* | 0.00 | 0.26 | 0.00 | 0.00 | 0.00 |
| *Lepidium draba* | 0.00 | 0.00 | 0.47 | 0.00 | 0.00 |
| *Lepidium draba* (2^nd^ pop) | 0.00 | 0.00 | 0.38 | 0.00 | 0.00 |
| *Lepidium latifolium* | 0.00 | 0.00 | 0.09 | 0.00 | 0.00 |
| *Lepidium latifolium* (2^nd^ pop) | 12.79 | 0.00 | 0.23 | 0.00 | 0.00 |
| *Lepidium squamatum* | 0.00 | 0.05 | 0.00 | 1.22 | 1.24 |
| *Lepidium squamatum* (2^nd^ pop) | 0.00 | 0.00 | 0.00 | 0.89 | 0.38 |
| *Stanleya pinnata* | 0.00 | 0.00 | 1.61 | 0.00 | 0.00 |
| *Stanleya viridiflora* | 16.24 | 0.00 | 0.85 | 0.00 | 0.00 |

| **Table S4 Continued** | | | | | |
| --- | --- | --- | --- | --- | --- |
|  | 6-(methylsulfinyl)hexyl | 2-methoxybenzyl and 3-methoxybenzyl | butenyl | benzyl | 4-methylthiobutyl |
| *Barbarea orthoceras* | 0.00 | 56.74 | 0.00 | 0.00 | 0.00 |
| *Brassica nigra* | 0.00 | 0.00 | 0.00 | 0.00 | 0.00 |
| *Brassica nigra* (2^nd^ pop) | 0.00 | 0.00 | 0.00 | 0.00 | 0.00 |
| *Camelina microcarpa* | 0.00 | 0.00 | 0.00 | 0.00 | 0.00 |
| *Camelina microcarpa* (2^nd^ pop) | 0.00 | 0.00 | 0.00 | 0.00 | 0.00 |
| *Caulanthus inflatus* | 0.00 | 0.00 | 0.00 | 0.54 | 0.00 |
| *Draba nemorosa* | 0.00 | 0.00 | 0.00 | 0.00 | 0.00 |
| *Hesperis matronalis* | 0.00 | 0.00 | 0.00 | 0.00 | 0.00 |
| *Lepidium campestre* | 0.54 | 0.00 | 0.00 | 0.00 | 0.00 |
| *Lepidium campestre* (2^nd^ pop) | 0.61 | 0.00 | 0.00 | 0.00 | 0.13 |
| *Lepidium crenatum* | 0.00 | 0.00 | 29.71 | 1.40 | 0.00 |
| *Lepidium draba* | 0.00 | 0.00 | 0.00 | 0.00 | 1.12 |
| *Lepidium draba* (2^nd^ pop) | 0.00 | 0.00 | 0.00 | 0.00 | 0.00 |
| *Lepidium latifolium* | 0.00 | 0.00 | 0.00 | 9.99 | 0.00 |
| *Lepidium latifolium* (2^nd^ pop) | 0.00 | 0.00 | 0.00 | 33.01 | 0.00 |
| *Lepidium squamatum* | 0.00 | 0.00 | 0.00 | 0.00 | 0.00 |
| *Lepidium squamatum* (2^nd^ pop) | 0.00 | 0.00 | 0.00 | 0.00 | 0.00 |
| *Stanleya pinnata* | 0.00 | 0.00 | 3.10 | 0.00 | 0.00 |
| *Stanleya viridiflora* | 0.00 | 0.00 | 8.08 | 1.96 | 0.00 |

| **Table S4 Continued** | | | | | | |
| --- | --- | --- | --- | --- | --- | --- |
|  | 3,4-dimethoxybenzyl | indolyl-methyl | unknown | 2-phenylethyl | 4-methoxyindol-3-ylmethyl | 9-(methylsulfonyl)nonyl and 9-(methylsulfinyl)nonyl |
| *Barbarea orthoceras* | 0.00 | 11.55 | 0.00 | 11.69 | 0.00 | 0.00 |
| *Brassica nigra* | 0.00 | 0.49 | 0.00 | 0.42 | 0.00 | 0.00 |
| *Brassica nigra* (2^nd^ pop) | 0.00 | 0.62 | 0.00 | 2.74 | 0.00 | 0.00 |
| *Camelina microcarpa* | 0.00 | 0.00 | 0.00 | 0.00 | 0.00 | 0.00 |
| *Camelina microcarpa* (2^nd^ pop) | 0.00 | 0.00 | 0.00 | 0.00 | 0.00 | 0.00 |
| *Caulanthus inflatus* | 0.00 | 0.21 | 0.00 | 0.00 | 0.57 | 0.00 |
| *Draba nemorosa* | 0.00 | 0.00 | 0.00 | 0.00 | 2.12 | 16.05 |
| *Hesperis matronalis* | 0.00 | 0.00 | 0.00 | 0.00 | 0.16 | 0.00 |
| *Lepidium campestre* | 0.00 | 0.00 | 0.00 | 0.00 | 0.94 | 0.00 |
| *Lepidium campestre* (2^nd^ pop) | 0.00 | 0.23 | 0.00 | 0.00 | 2.15 | 0.00 |
| *Lepidium crenatum* | 0.00 | 0.00 | 0.00 | 0.00 | 0.31 | 0.00 |
| *Lepidium draba* | 0.00 | 0.14 | 0.00 | 0.00 | 1.54 | 0.00 |
| *Lepidium draba* (2^nd^ pop) | 0.00 | 0.18 | 0.00 | 0.00 | 0.99 | 0.00 |
| *Lepidium latifolium* | 0.00 | 0.00 | 0.00 | 0.00 | 1.56 | 0.00 |
| *Lepidium latifolium* (3^rd^ pop) | 0.00 | 0.00 | 0.00 | 0.00 | 2.12 | 0.00 |
| *Lepidium squamatum* | 0.32 | 0.00 | 9.06 | 0.00 | 0.00 | 0.00 |
| *Lepidium squamatum* (2^nd^ pop) | 0.52 | 0.00 | 10.93 | 0.00 | 0.14 | 0.00 |
| *Stanleya pinnata* | 0.00 | 0.00 | 0.00 | 0.00 | 0.00 | 0.00 |
| *Stanleya viridiflora* | 0.00 | 3.04 | 0.00 | 0.00 | 1.81 | 0.00 |

Figure S1a. Dendrograms for species tested based on GS profiles from all leaves, young leaves only (collected above the middle node), and old leaves only (collected below the middle node) and using all 13 species.

Figure S1b. Dendrograms for species tested based on GS profiles from all leaves, young leaves only (collected above the middle node), and old leaves only (collected below the middle node) and using the species tested with *P. xylostella*.
